# Mönckeberg's Medial Calcific Sclerosis Makes Traditional Arterial Doppler's Unreliable in High-Risk Patients with Diabetes

**DOI:** 10.1177/15347346231191588

**Published:** 2023-07-31

**Authors:** Mehmet A. Suludere, Sahab K. Danesh, Amanda L. Killeen, Peter A. Crisologo, Matthew Malone, Michael C. Siah, Lawrence A. Lavery

**Affiliations:** 1 Department of Plastic Surgery, 12334 University of Texas Southwestern Medical Center, Dallas, Texas, USA; 2 Infectious Diseases and Microbiology, School of Medicine, Western Sydney University, Liverpool, New South Wales, Australia; 3 Department of Vascular Surgery, 12334 University of Texas Southwestern Medical Center, Dallas, Texas, USA

**Keywords:** Mönckeberg's sclerosis, diabetic foot, ankle-brachial index, noncompressible arteries, arterial Doppler

## Abstract

**Objective:**

To assess Mönckeberg's medial calcific sclerosis (MMCS) severity in patients with a diabetic foot infection.

**Methods:**

This was an analysis of 2 randomized clinical trials in which we evaluated the treatment of 233 patients admitted to the hospital for moderate and severe foot infections. Arterial calcification was defined as visible radiopaque arteries on foot and ankle radiographs, recorded as the most distal visible artery involved (toes, metatarsals, and ankle/hindfoot).

**Results:**

Most subjects (57.1%, *n* = 133) had MMCS, with extension to toes in 79 (59.4%), to metatarsals in 32 (24.1%), and to ankle/hindfoot in 22 patients (16.5%). In 7 patients (5.2%) MMCS was solely seen in dorsalis pedis (DP) artery, in 13 patients (9.8%) in posterior tibialis (PT) artery, and in 113 patients (85.0%) MMCS was seen in both arteries. Only 29.2% (*n* = 68) of DP arteries and 34.8% (n = 81) of PT arteries were not compressible by Doppler. DP and PT arteries were not compressible more often in MMCS (DP 34.3% vs 20.4%, *P *= .02 and PT 43.1% vs 21.4%, *P *< .01), toe-brachial indices of ≥0.7 were significantly more common in people without MMCS (46.0% vs 67.4%, *P *< .01). In contrast, there were no differences in skin perfusion pressure measurements (>50 mmHg; 67.7% vs 68.0%, *P *= .96), waveforms (biphasic/triphasic 83.5% vs 77.0%, *P *= .22), and pulse volume recording (9.6 ± 3.3 vs 13.7 ± 36.0) between patients with and without MMCS.

**Conclusion:**

MMCS is common in patients with diabetic foot infections. MMCS is associated with noncompressible arterial Doppler studies and likely interferes with the accuracy of arterial Doppler studies.

## Introduction

Mönckeberg's medial calcific sclerosis (MMCS) is a calcification of the tunica media of medium-sized arteries, commonly visible on plain film radiographs. It is often seen in persons with diabetes, peripheral arterial disease (PAD), the elderly, and patients with chronic kidney disease with end-stage renal disease.^1–3^ Arterial calcification leads to loss of elasticity of the vessel wall, causing vascular stiffening and decreased compliance of the vessel.^2^ MMCS is most observed in the feet and progresses proximally.^4^ Systolic foot and ankle pressures, ankle-brachial indices (ABIs), and toe-brachial indices (TBIs) are common noninvasive studies to assess arterial disease in addition to the healing potential of wounds.^5^ Calcification of the arterial vessels leads to falsely elevated or noncompressible vessels, which leads to unreliable values.^6,7^

The prevalence of MMCS has been reported in the literature in different risk groups, however, the extent of MMCS in the foot and its association with noncompressible arterial Doppler studies and abnormal waveforms has not been reported. The purpose of this study was to evaluate the prevalence and extent of Mönckeberg's sclerosis in persons with diabetes admitted to the hospital for moderate and severe diabetic foot infections. The study aimed to compare systolic pressures, waveforms, and skin perfusion pressure (SPP) measurements in subjects with and without MMCS.

## Methods

This study is a prospective cohort study that utilized the data collected from 2 randomized clinical studies published by our research group.^8,9^ A combined data set was used for the post hoc analysis. The 2 randomized clinical studies compared negative pressure wound therapy with and without saline irrigation in complex foot infections. Patients were consented, and approval from the Ethics committee was obtained. Data collected in the studies included broad demographics, comorbidities, history of drug, alcohol, or tobacco use, foot ulcer history, foot infection history, amputation history, sensory neuropathy, labs drawn during admission, and peripheral vascular surgery history.

The inclusion criteria were moderate or severe foot infection, ABI ≥ 0.5 or toe pressures >30 mmHg, age ≥18 years. The exclusion criteria were active Charcot arthropathy, malnutrition (defined as body mass index < 19), untreated bone or soft tissue infection, active alcohol or substance abuse, active malignancy or melanoma at the wound site, sepsis (defined as positive blood culture with leukocytosis), HIV, and developmental disability/significant psychological disorder.

In both previous studies, infection severity was defined using criteria from the International Working Group on the Diabetic Foot—Diabetic Foot Infection guidelines.^10–12^ Diabetes was defined using criteria from the American Diabetes Association.^13^ End-stage renal disease (ESRD) was defined as an estimated glomerular filtration rate <15 mg/mmol or a patient undergoing dialysis. Vascular data included systolic pressures at the posterior tibial (PT) and dorsalis pedis (DP) arteries, and the great toe, dorsal, and plantar SPP measurements (Sensilase, Vasamed, Eden Prairie, MN), waveforms, and pulse volume records (PVRs). PAD was defined as ABI < 0.9. Noncompressible vessels were defined as ABI ≥ 1.3. Normal SPP was defined as created than ≥50 mmHg.^14–16^ Sensory neuropathy was defined as vibration perception threshold >25 Hz at the great toe and medial malleolus (Biothesiometer, Xilas Medical, San Antonio, TX), or any sampled site missed with a 10 g Semmes Weinstein monofilament.^17,18^ Arterial calcification was defined as visible radiopaque arteries identified on foot and ankle radiographs (anterior posterior and lateral views). The location of visible calcification was collected as the most distal visible artery on an ordinal scale. The location of the calcification was categorized as follows: toes (distal to the metatarsophalangeal joint), metatarsals (metatarsophalangeal joint to Lis Franc joint), ankle/hindfoot (the navicular-cuneiform joint to ankle joint), and no calcification.

The primary outcome of interest was the prevalence of MMCS in the foot. Secondary outcomes included comparing systolic pressures, ABIs calculated from both the PT and DP arteries, TBIs calculated from the pressures of the big toe (not calculated if the patient did not have a big toe), pulse volume recordings, and SPP in patients with and without MMCS.

Descriptive statistics were performed with Statistical Package for Social Sciences (SPSS) version 27. We presented study variables as medians, means, and standard deviations (SDs) for continuous variables and proportions or percentages for dichotomous variables. We reported odds ratios (OR) and 95% confidence intervals (CIs). For comparing dichotomous variables, we used the chi-squared test. We considered an alpha of 0.05 as the threshold for significance.

## Results

**Figure 1.**
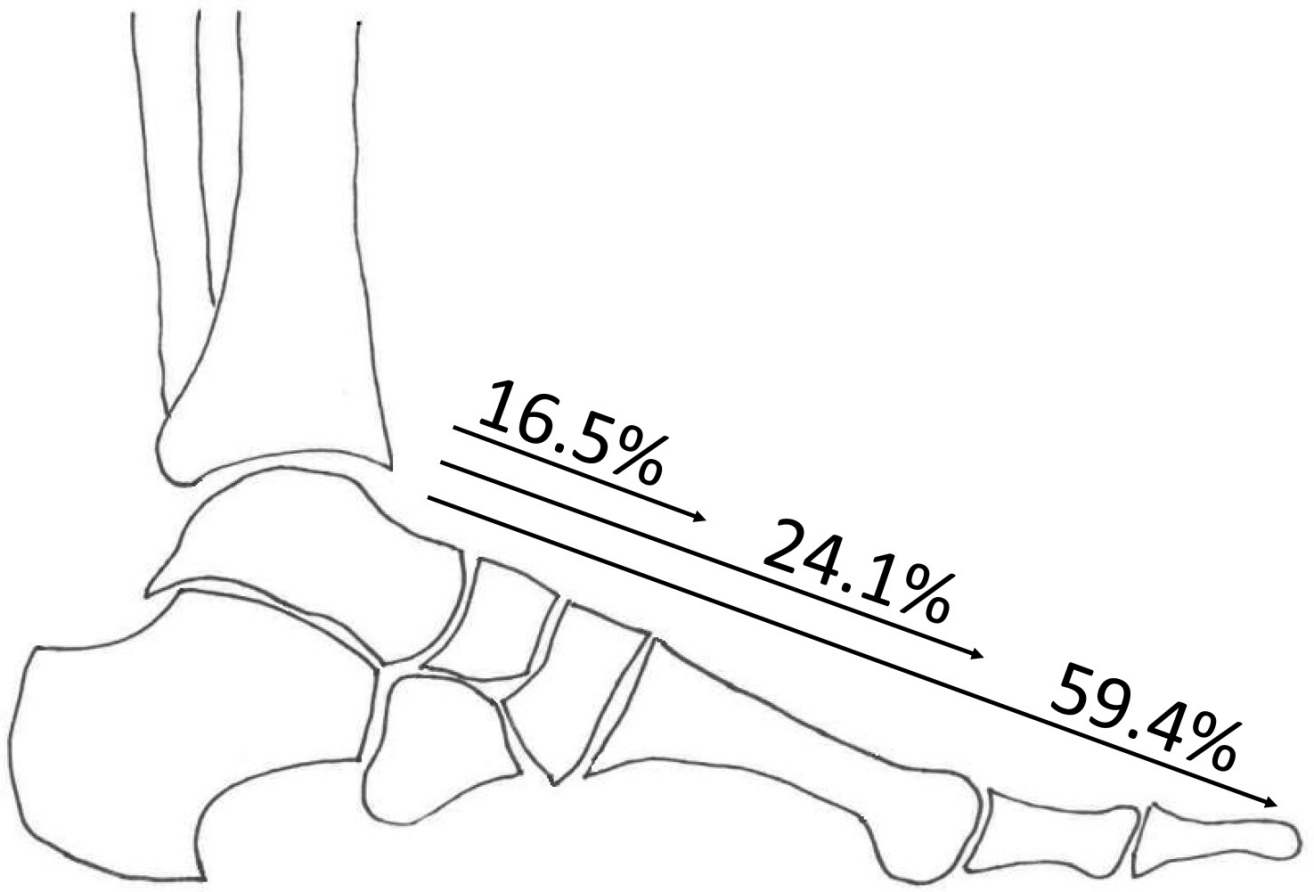
This figure shows the extension of calcification in the arteries of the foot in people with Mönckeberg's sclerosis. Among patients with Mönckeberg's, calcification extends to the ankle in 16.5% of patients, to the metatarsals in 24.1% of patients, and to the toes in 59.4% of subjects.

In total, 233 patients with diabetes were included in the analysis. The majority of study subjects (57.1%, *n *= 133) had MMCS. MMCS extended to the toes in 79 patients (59.4%), to the metatarsals in 32 patients (24.1%), and to the ankle/hindfoot in 22 patients (16.5%), as shown in Figure 1. In 7 patients (5.2%), MMCS was solely seen in the DP, in 13 patients (9.8%) in the PT artery, and in 113 patients (85.0%) MMCS was seen in both arteries. Patients with abnormal vibration perception threshold, longer duration of diabetes, and ESRD were significantly more likely to have MMCS (Table 1).

**Table 1.** Patient Demographics, Comorbidities, and Past Medical History.

	Mönckeberg's (*n* = 133)	No Mönckeberg's (*n* = 100)	Odds ratio (95% CI)	*P*-value
Male	108 (81.2)	76 (76)	1.4 (0.7-2.6)	.93
Age	51.7 ± 9.6	49.5 ± 9.4	(−0.2 to 4.7)	.08
BMI (kg/m^2^)	32.09 ± 7.4	31.5 ± 7.8	(−1.4 to 2.6)	.57
Race				
Caucasian	16 (12.0)	36 (36.0)	0.2 (0.1-0.5)	<.01
African American	40 (30.1)	32 (32.0)	0.9 (0.5-1.6)	<.01
Hispanic	75 (56.4)	32 (32.0)	2.8 (1.6-4.7)	<.01
Native American	2 (1.5)	0 (0)	3.8 (0.2-80.5)	.39
Diabetes Type 2	127 (95.5)	95 (95.0)	1.3 (0.4-4.7)	.65
Diabetes duration (years)	17.1 ± 8.8	12.0 ± 6.8	(3.0-7.2)	<.01
Substance abuse				
Tobacco	68 (51.1)	54 (54.0)	0.9 (0.5-1.5)	.67
Alcohol	75 (56.4)	57 (57.0)	1.0 (0.6-1.7)	.93
Illicit drugs	26 (19.6)	17 (17.0)	1.7 (0.9-3.4)	.62
Foot ulcer history				
Study foot	36 (26.1)	35 (35.0)	0.7 (0.4-1.2)	.19
Contralateral foot	25 (18.8)	14 (14.0)	1.4 (0.7-2.9)	.33
Both feet	23 (17.3)	14 (14.0)	1.2 (0.6-2.6)	.46
Foot infection history	79 (59.4)	66 (66.0)	0.8 (0.4-1.3)	.30
Amputation history	60 (45.1)	41 (41.0)	1.2 (0.7-2.0)	.53
Cardiac angioplasty	9 (6.8)	2 (2.0)	3.6 (0.8-16.4)	.12
Coronary artery bypass	3 (2.3)	3 (3.0)	2.2 (0.6-0.86)	1.0
Myocardial infarction	8 (6.0)	6 (6.0)	1.0 (0.3-3.0)	.10
Retinopathy	33 (24.8)	12 (12.0)	2.4 (1.2-5.0)	.14
Peripheral vascular surgery history	11 (8.3)	2 (2.0)	4.4 (1.0-20.4)	.04
Congestive heart failure	18 (13.5)	7 (7.0)	2.1 (0.8-5.2)	.11
Stroke	5 (3.8)	3 (3.0)	1.3 (0.3-5.4)	1.0
Chronic kidney disease				
Stages 1–4	43 (32.3)	21 (21)	1.8 (1.0-3.3)	.06
End-stage renal disease	14 (10.5)	0 (0)	24.4 (1.4-414.0)	.03
Sensory neuropathy (SWM or VPT)	132 (99.2)	99 (99.0)	1.3 (0.1-21.6)	.84
Abnormal monofilament	126 (94.7)	95 (95.0)	1.06 (0.3-3.4)	.71
Abnormal VPT	124 (93.2)	85 (85.0)	0.41 (0.2-1.0)	.04
Glycated hemoglobin	10.0 ± 3.5	9.9 ± 2.7	0 (−0.8 to 0.7)	.95

Abbreviations: BMI, body mass index; SWM, Semmes Weinstein monofilament; VPT, vibration perception threshold; SD, standard deviation. Dichotomous variables are presented as *n* (%). Continuous variables are presented as mean ± SD. Chi-square or Fisher's exact test performed to compare dichotomous variables.

The effect of MMCS on vascular studies is shown in Table 2. It is often assumed that noncompressible arteries during Doppler examinations are due to MMCS. In the whole study population, 40.3% (*n* = 94) of the subjects had at least 1 artery that was not compressible with arterial Doppler. In patients with MMCS (*n* = 133), 34.3% of DP and 43.1% of PT arteries were not compressible during arterial Doppler examination (Table 2). A greater number of DP and PT arteries were not compressible in people with MMCS when compared to those without MMCS (DP: 34.3% vs 20.4%, *P *= .02; PT: 43.1% vs 21.4% *P *< .01). Conversely, normal ABIs (0.9-1.29) were significantly more common in people without arterial calcification on radiographs (ABI PT: 47.4% vs 72.8%, *P *< .01; DP: 57.7% vs 74.8%, *P *= .01). For the analysis of the TBIs, we included 212 patients (no MMCS 40.6% vs 59.4% with MMCS) as 21 patients did not have big toes due to previous amputation. In the patients with TBIs, MMCS was significantly more common in patients with TBIs ≥ 0.7 (no MMCS 46.0% vs MMCS 67.4%, *P *< .01).

**Table 2.** Vascular Study Parameters in MMCS.

	Mönckeberg's (*n* = 133)	No Mönckeberg's (*n* = 100)	*P*-value
ABI			
DP artery			
≥1.30	47 (34.3)	21 (20.4)	.02
0.90-1.30	79(57.7)	77(74.8)	.01
<0.90	11 (8.0)	5 (4.9)	.33
Posterior tibialis artery			
≥1.30	59 (43.1)	22 (21.4)	<.01
0.90-1.30	65 (47.4)	75 (72.8)	<.01
<0.90	13 (9.5)	6 (5.8)	.30
Toe-brachial index (*n* = 212)	0.7 ± 0.3	0.8 ± 0.3	.42
≥0.50	93 (73.8)	66 (76.7)	.62
≥0.70	58 (46.0)	58 (67.4)	<.01
Pulse volume recording (mmHg)	9.6 ± 3.3	13.7 ± 36.0	.40
Waveform biphasic/triphasic	111(83.5)	77 (77.0)	.22
SPP >50 mmHg			
Dorsal lateral	92 (70.8)	73 (75.3)	.45
Dorsal medial	88 (67.7)	66 (68.0)	.96
Plantar lateral	110 (87.3)	90 (92.8)	.18
Plantar medial	111(86.0)	86 (88.7)	.56

Abbreviations: MMCS, Mönckeberg's medial calcific sclerosis; ABI, ankle-brachial index; DP, dorsalis pedis; SPP, skin perfusion pressure; SD, standard deviation. Dichotomous variables are presented as *n* (%). Continuous variables are presented as mean ± SD. Chi-square or Fisher's exact test was performed to compare dichotomous variables.

In contrast, there were no statistical differences in SPP measurements (>50 mmHg, 67.7% in MMCS vs 68.0%, *P *= .96), waveforms (biphasic/triphasic: 83.5% vs 77.0%, *P *= .22), and pulse volume recording (9.6 ± 3.3 vs 13.7 ± 36.0) in patients with MMCS and without MMCS, respectively (Table 2).

## Discussion

Our results show a high prevalence of MMCS (57.1%) in patients admitted to the hospital with moderate and severe diabetic foot infections. To the best of our knowledge, no previous study has compared abnormalities in ABIs, TBIs, SPP, waveforms, and PVRs in patients with and without MMCS. The prevalence of MMCS was much higher than the rate of noncompressible ABIs using arterial Dopplers. Approximately 30% of the arteries could not be assessed by Doppler because they were not compressible. MMCS may decrease the reliability of ABIs; however, even in patients with no MMCS, ABIs of both the DP and posterior tibial arteries were not compressible in about 20% of arteries with no visible calcification on radiographs. Artery stiffness makes the arterial Doppler approach suspect. One of the interesting findings in this study were the similar measurements in SPP in patients with and without MMCS. SPP measurements in this study suggested that most subjects with MMCS had normal skin perfusion. SPP and PVRs have been reported to be better predictors of wound healing than ABIs.^19^ Diagnostic methods that are not affected by the compressibility of arteries such as SPP or hyperspectral imaging may be more reliable to predict healing or PAD severe enough to impede healing. SPP and hyperspectral imaging have the advantage of allowing measurements on the dorsum and sole of the foot. Because SPP has been commercially available longer, there are larger studies that evaluate different cut-off points to predict wound healing. A meta-analysis by Pan et al^20^ studied the predictive value of SPP in wound healing, they found a pooled sensitivity of 79.9% (95% CI, 73.9%-84.9%) and a pooled specificity of 78.2% (95% CI, 61.5%-89.0%) for 30 mmHg as a cut-off point, and a pooled sensitivity of 67.1% (95% CI, 55.8%-76.8%) and a pooled specificity of 84.2% (95% CI, 74.0%-90.9%) for 40 mmHg as a cut-off point.^20^ But the device is expensive, it is not easy to move to different settings, and it is not available in many hospitals. Furthermore, the SPP system that is used can affect the results. The type, size, and shape of the probe influences the SPP measurements.^21,22^ There are several hyperspectral imaging devices that offer point and shoot technology, so capturing data is very fast. This allows data to be captured quickly, and the technology can be used in clinics as easily as it can be used in the hospital or operating room. There is no robust data to show cut-offs to predict healing with hyperspectral imaging.^23,24^

The prevalence of MMCS is influenced by other conditions that often occur together with diabetes. This results in high variances in the literature. The prevalence of MMCS in the lower extremity is reported to be between 17% in newly diagnosed people with diabetes and as high as 92% in persons with diabetes or chronic kidney disease who had a lower extremity amputation.^3,4,25–27^ The MMCS prevalence results in this study are similar to studies that evaluate high-risk subpopulations, such as those reported by Aragón-Sánchez et al^1^ who reported 59.4% prevalence of MMCS in their population of patients with diabetes admitted for acute foot disease. Young et al^28^ examined the prevalence of MMCS in 4 groups: nondiabetic controls (22.5%), diabetics without neuropathy (25.0%), diabetics with neuropathy (61.9%), and diabetics with neuropathy and history of foot ulcer (78.8%).

The highest prevalence of MMCS was reported by Lew et al.^25^ They identified radiographic artery calcification of 92.1% in patients who underwent a lower extremity amputation.^25^ The prevalence of MMCS they identified was much higher than our findings. This could be due to several factors. Firstly the difference could be explained by their study population. It consisted of patients with PAD, diabetes mellitus, and chronic kidney disease who were all in the hospital for lower extremity amputation. Besides this, they used plain radiography or computed tomography angiography (CTA) to demonstrate the presence of medial arterial calcification, this can lead to observing more patients with arterial calcification than with plain radiography only, as CTA is more susceptible to trace calcifications.^29^ Nevertheless, their results suggest that there is more calcification present than our study results showed. While it might be true that there is overdiagnosis of medial arterial calcification, it is likely that the truth may be somewhere in the middle.

There are several limitations to our study. The data from this analysis was from a post hoc evaluation of data from 2 randomized clinical studies. We excluded patients with active Charcot foot and patients with an ABI < 0.5 or toe pressures <0.30 mmHg. The study included persons with moderate and severe diabetic foot infections which required hospitalization and surgery. Clearly there was selection bias based on inclusion and exclusion criteria. In addition, we were not able to separately identify intimal and medial vessel calcification which might lead to measurement bias. Although both can be seen on plain film radiographs as vessel calcification, intimal calcification affects blood flow inside the vessel lumen and medial calcification only affects the vessel wall. This could affect the results seen in the ABIs as intimal calcification would narrow or block the vessel lumen changing the waveform, while medial calcification would cause the vessel to be less compliant or noncompressive. We also observed arteries that were not compressible without calcifications on radiographs. Furthermore, we realize that some of the arterial calcifications we measured may not be associated with MMCS, while we also think that some of the MMCS present in the arteries may not have been visible on the foot and ankle radiographs.

## Conclusion

This study reports the prevalence and extent of Mönckeberg's sclerosis in patients with moderate and severe diabetic foot infections. Mönckeberg's sclerosis was identified in 57.1% of the patients; however, considerably fewer subjects had noncompressible arterial vessels. This suggests that observations of arterial calcification on radiographs may represent a spectrum of arterial stiffness. Technology that does not rely on compressibility of the arteries of the foot such as SPP and hyperspectral imaging may be more accurate than traditional arterial Dopplers since MMCS may render arterial pressures in the foot and ankle may be unreliable in the majority of patients. Further work is needed to understand the role of MMCS in assessing wound healing.
